# Post-Deployment Screening of Thailand Military Units Deployed to South Sudan from 2023 to 2025 Reveals High Rates of Sub-Microscopic *P. falciparum* Malaria

**DOI:** 10.3390/tropicalmed11060159

**Published:** 2026-06-15

**Authors:** Min Kramyoo, Sidhartha Chaudhury, Watcharee Yokanit, Kamonwan Siriwattanakul, Porruthai Kittikanara, Brian A. Vesely, Darunee Utennam, Nakarin Sansanayudh, Sutchana Tabprasit

**Affiliations:** 1 Research Division, Armed Forces Research Institute of Medical Sciences, Bangkok 10400, Thailand; 2 Walter Reed Army Institute of Research-Armed Forces Research Institute of Medical Sciences, Bangkok 10400, Thailand

**Keywords:** *Plasmodium*, exposure, parasitemia, prophylaxis failure, screening, peacekeeping missions

## Abstract

Peacekeeping operations in sub-Saharan Africa continue to be impacted by malaria both in-country and among returning service members. The Royal Thai Army (RTA) deploys an engineering company to Juba and Rumbek, South Sudan to conduct peacekeeping operations as part of the UN Mission in South Sudan (UNMISS). Each deployment is approximately 12 months long. The unit is given doxycycline one week before travel before switching to the UN-provided mefloquine during deployment and for four weeks after returning. The RTA routinely conducts post-deployment screening for malaria by microscopy and PCR for units returning from UNMISS. High rates of prophylaxis failure were observed from both during-mission and post-deployment screening cases, with cumulative malaria attack rates of 11.4% (31 cases out of 271 personnel), 18.2% (49 cases out of 270 personnel), and 23.1% (63 cases out of 273 personnel) for 2023, 2024, and 2025, respectively, with 98% of cases being due to *P. falciparum*. Furthermore, post-deployment screening revealed high rates of sub-microscopic and sub-clinical parasitemia with 40% of all malaria cases being identified as asymptomatic during post-deployment screening, and 61% of those asymptomatic cases being detected by PCR only. While factors underlying the high prophylaxis failure rate, as well as the high rate of sub-microscopic and sub-clinical parasitemia are unclear, these findings highlight the limitations of relying on clinical symptoms or microscopy for detecting malaria in military units returning from endemic regions and underscore the importance of unit-wide post-deployment molecular screening.

## 1. Introduction

Malaria remains a persistent public health threat to those living in endemic regions as well as to military forces deployed to those areas. Since 2018, Royal Thai Army (RTA) military personnel have been annually deployed to South Sudan for peacekeeping operations as part of the UN Mission in South Sudan (UNMISS). Like several sub-Saharan African countries, South Sudan is an area with an extremely high malaria disease burden. In 2024, South Sudan reported 237.6 per 1000 population of malaria case incidence at risk [1], with *Plasmodium falciparum* accounting for 93.1% of cases [2]. Despite the advent of numerous chemoprophylaxis drugs, prophylaxis failure remains a significant problem for military units deploying to sub-Saharan Africa for peacekeeping missions or stabilization operations [3,4,5,6,7,8].

The three most commonly prescribed medications for chemoprophylaxis for malaria are atovaquone–proguanil, doxycycline, and mefloquine [9,10,11]. Historically, deploying military units was usually given the WHO-recommended daily dosing of doxycycline [12] as a chemoprophylaxis based both on its efficacy and its cost-effectiveness. In recent years, daily atovaquone–proguanil (Malarone™) dosing and weekly mefloquine dosing have seen increased military usage due to fewer side effects and/or improved compliance [13] that is thought to be essential for long-term chemoprophylaxis [11].

Despite the use of chemoprophylaxis, peacekeeping and stabilization operations continue to be impacted by malaria in endemic regions. In some cases, this malaria impact is represented by acute cases during the deployment, usually of *P. falciparum*, that is the result of prophylaxis failure. In 1995–1996, a Brazilian military unit of 1200 personnel on a peacekeeping mission in Angola experienced 78 cases of acute *P. falciparum* malaria, including four hospitalizations and three deaths, despite being provided mefloquine prophylaxis. In 2003, 44 U.S. marines out of a unit of 225 personnel that were deployed to Liberia in response to civil unrest needed to be evacuated due to *P. falciparum* malaria [8] despite also being given mefloquine. Mefloquine prophylaxis failure has also been reported by UN peacekeeping forces in the Democratic Republic of the Congo (DRC) in 2010 [14] and the Central African Republic (CAR) in 2016–2022 [15,16]. In other cases, the impact of malaria on military operations is reflected in latent cases that emerge weeks to months after deployment, due to the fact that while chemoprophylaxis can suppress or prevent acute blood-stage parasitemia, it cannot prevent the establishment of latent liver-stage infection, such as with *P. vivax* and *P. ovale*. A retrospective analysis of French soldiers returning from the Ivory Coast from 1993 to 2012 identified a cumulative total of 295 cases of *P. ovale* malaria in units that were given daily doxycycline prophylaxis [4]. More recent examples involving latent malaria cases following mefloquine prophylaxis include *P. ovale* cases in UN peacekeeping missions in the DRC [5], CAR [3], and South Sudan [7,17].

Traditionally, military units operating in austere environments rely on two WHO-recommended methods for malaria diagnosis: microscopy or rapid diagnostic tests (RDTs). Microscopy provides a sensitive, highly specific diagnosis of malaria that allows for parasite quantification and speciation. A skilled microscopist can detect asexual parasites at a density of <10 per µL of blood, but under typical field conditions, the limit of sensitivity is approximately 100 parasites per µL [18]. However, microscopy involves relatively high costs for training and supervision, and its accuracy is strongly dependent on the competence of the microscopist. Malaria RDTs are immuno-chromatographic tests for detecting parasite-specific antigens in a finger-prick blood sample. The test provides rapid results and can extend diagnostic capabilities to the lowest-level health facilities and communities with fewer requirements for training and skilled personnel. However, these RDTs are designed to reliably detect malaria at parasite densities of ≥200 parasites/µL and for malarias with lower parasite densities, such as *P. vivax* or *P. ovale*, RDTs often show poor sensitivity [19]. Real time PCR techniques to detect malaria have been shown to be both highly sensitive and highly specific [20,21], even at very low parasite densities (<0.1 parasites per µL) [22,23]. While useful for specialized epidemiological investigations, they are not generally available for field use in malaria- endemic areas.

While the diagnosis of symptomatic malaria is critical for effective clinical management, asymptomatic sub-patent infections have received comparatively less attention [24,25]. This lack of focus is partly due to the challenges associated with detecting asymptomatic infections, which often require active surveillance and molecular diagnostic techniques [25]. The prevalence of asymptomatic *Plasmodium* infections can vary significantly from an estimated 5.8% in Southeast Asia [26] to as high as 40% among high-risk populations in Sub-Saharan Africa [27,28]. Asymptomatic, pre-clinical, or sub-patent malaria cases in service members returning from deployments in endemic regions can lead to the importation of malaria to their home country [24,26]. These cases can also potentially develop into symptomatic malaria once the chemoprophylaxis regimens used during deployment are halted and their prophylactic effects have lapsed [17]. As such, implementation of screening methods for asymptomatic malaria in military personnel is of critical importance. The RTA routinely conducts post-deployment screening for malaria in units returning from endemic regions. Here, we present recent findings from the RTA UNMISS deployments from 2023 to 2025 in order to better understand the disease burden of malaria during these deployments and assess and evaluate strategies for surveillance.

## 2. Materials and Methods

All returning personnel underwent mandatory malaria screening within 24 h of arrival in Thailand as part of routine public health surveillance. This screening consisted of a venous blood draw and a questionnaire but did not include a physical examination. At the time of screening, none of the individuals reported symptoms or showed clinical signs consistent with malaria, including fever. A total of 914 samples were included in the analysis, comprising 271 samples collected in 2023, 370 in 2024, and 273 in 2025. All laboratory and epidemiological data presented here were collected as part of routine medical screening and was anonymized and recorded using coded identifiers prior to analysis, with no personal identifiers linked to individual participants.

Venous blood samples were collected in ethylenediaminetetraacetic acid (EDTA) tubes. Thick and thin blood films were prepared immediately and stained with Giemsa following standard procedures. Microscopic classification was made using two independent microscopists and any discrepancies were resolved by a third independent microscopist. Thick films were used for parasite detection, while thin films were examined for *Plasmodium* species identification and parasite morphology. For real-time PCR testing, DNA was extracted from whole blood samples using the Beaverbeads DNA/RNA Kit (Kingsmead Service B.V., Nootdorp, The Netherlands), and then PCR detection was conducted using the VIASURE Malaria differentiation Real Time PCR Detection Kit (CerTest Biotec, Zaragoza, Spain). The multiplex real-time PCR assay included genus-specific targets, followed by species-specific assays targeting five human malaria parasites (*P. falciparum*, *P. vivax*, *P. malariae*, *P. ovale*, and *P. knowlesi*). Appropriate positive and negative controls were included in each PCR run.

Descriptive statistics were used to summarize screening results. Asymptomatic infections were defined as any case where an individual was malaria positive but reported no symptoms, typically found during post-deployment unit-wide screening. Sub-microscopic cases were defined as individuals that were negative for malaria by microscopy but positive by PCR. Diagnostic performance of microscopy in terms of sensitivity and specificity was evaluated in comparison with real-time PCR. Agreement between microscopy and real-time PCR results was assessed using Cohen’s kappa coefficient (κ).

## 3. Results

**Figure 1:**
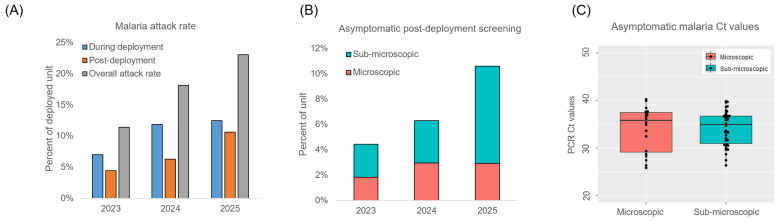
(**A**) Malaria attack rates increased for in-country (blue), post-deployment screening (orange), and total (gray) for UNMISS deployments in 2023, 2024, and 2025. (**B**) Post-deployment asymptomatic screening results for sub-microscopic (PCR positive only, blue) and microscopic (microscopy positive, pink) following deployments in 2023, 2024, and 2025. (**C**) PCR Ct values from microscopic (pink), and sub-microscopic (blue) malaria positive cases identified during asymptomatic post-deployment screening.

A total of 271, 270, and 273 personnel were deployed to South Sudan in 2023, 2024, and 2025, respectively. All deployments were approximately 12 months in duration. Malaria attack rates increased steadily from 2023 to 2025 across all measured categories (Figure 1A). The total number of malaria cases detected in-mission increased over the three years from 19 cases in 2023 to 32 cases in 2024 and 34 cases in 2025, corresponding to in-mission attack rates of 7.0%, 11.8%, and 12.4% over the three years. Post-deployment screening was conducted on asymptomatic individuals and demonstrated a similar upward trend, increasing from 12 cases in 2023 and 17 cases in 2024, to 29 cases in 2025. The proportion of asymptomatic cases over total cases has been relatively stable at 39%, 35%, and 46% over the three years. Overall, the total attack rate (in-mission combined with post-deployment cases) nearly doubled over the three-year period, rising from approximately 11% in 2023 to around 23% in 2025, indicating a progressive increase in malaria burden among deployed personnel (Table 1).

Post-deployment screening results further revealed a growing contribution of sub-microscopic infections over time (Figure 1B). A majority of cases identified during post-deployment screening were identified by PCR only, and thus classified as sub-microscopic and accounted for 7 cases in 2023, 9 cases in 2024, and increased markedly to 21 cases in 2025. Thus, sub-microscopic infections represented a substantial proportion of total cases across all years, accounting for 58% in 2023, 53% in 2024, and rising to 72% in 2025. In terms of overall numbers, microscopic detections increased from 1.8% in 2023 to 3.0% in 2024 and 2.9% in 2025. In contrast, sub-microscopic infections rose from 2.6% in 2023 to 3.3% in 2024, followed by a marked increase to 7.7% in 2025.

We assessed the Ct values from the PCR results to determine if sub-microscopic malaria was characterized by lower parasite density, and thus higher Ct values (Figure 1C). However, we found no significant difference in Ct values between microscopic and sub-microscopic cases suggesting that there was no substantial difference in parasite DNA levels between the two groups. Finally, we compared the sensitivity and specificity of microscopy compared to real-time PCR in detecting malaria during asymptomatic screening. We found that while microscopy had a high specificity of 100%, it had a low sensitivity of 37% with an overall Cohen’s κ of 0.51, indicating a moderate level of agreement.

## 4. Discussion

This study demonstrates a progressive increase in malaria cases detected among deployed personnel over the three-year period from 2023 to 2025. The rising overall attack rate and asymptomatic infection rate suggest ongoing exposure to malaria transmission during deployment, despite preventive measures. The causes underlying the rising attack rate from 2023 to 2025 are unclear. Malaria rates in South Sudan from 2023 to 2025 reported by the World Health Organization reflect only a modest increase from 2.8 m cases in 2023 to 3.0 m cases in 2025 [29,30,31]. It is possible that the exact locations and activities of the unit during the deployment in different years exposed them to different levels of malaria transmission. In any case, these findings underscore the continued risk of malaria among deployed personnel and the need to evaluate and update malaria surveillance and prevention strategies.

A key finding of this study is the substantial contribution of PCR-based diagnostics to case detection. Across all years, real-time PCR identified considerably more infections than microscopy, with sub-microscopic infections accounting for more than half of all cases. Although it is well-known that PCR is more sensitive than microscopy, the degree to which *P. falciparum* malaria can present as sub-microscopic in a largely *P. falciparum* malaria-naïve population under chemoprophylaxis has, to our knowledge, not previously been reported. The high proportion of asymptomatic infections further emphasizes the challenges with detecting malaria in this population. It is important to note that other studies have observed high sub-microscopic and asymptomatic malaria rates in military settings. A study conducted in 2019 with the Papua New Guinea Defense Force found that 87% of malaria cases were sub-microscopic, albeit mostly resulting from *P. vivax* infections [32]. Among the Brazilian unit that deployed to Angola, 11% were found to have asymptomatic malaria following post-deployment screening, mostly of *P. falciparum* [33]. In the present study, post-deployment screening proved to be an important component of malaria surveillance, identifying a substantial number of infections that may have otherwise been missed. The higher number of cases detected post-deployment compared with in-country diagnosis indicates that reliance on passive case detection alone is insufficient, particularly in settings where clinical symptoms may be mild or absent. Overall, these findings highlight the impact of integrating molecular methods into routine post-deployment screening.

Prior studies have highlighted the significant challenges in malaria prophylaxis failure among personnel deployed to endemic regions in Africa. Despite standardized chemoprophylaxis, attack rates had ranged from 6.4% in Thai peacekeepers returning from South Sudan [7] to a substantial 35% among U.S. Marines in Liberia [8]. These prophylaxis failures may be attributed to poor chemoprophylaxis compliance. For example, a retrospective study of UN troops deployed to Angola for peacekeeping missions reported that 46% of personnel who were given mefloquine prophylaxis had blood concentrations of the drug that were below the required prophylaxis level, including all personnel who were confirmed positive for malaria [6]. Other factors could include drug resistance [14,15], and the inability of standard blood-stage prophylaxis to clear liver-stage hypnozoites [3,4], leading to delayed relapses after returning to non-endemic countries [3,5,7].

The observed high sub-microscopic rates and asymptomatic rates may be caused by the suppressive effect of sub-therapeutic drug levels [6], which suppress parasite load enough to prevent a systemic inflammatory response but are unable to completely clear the infection [7,16]. This theory is corroborated by the relatively high PCR ct counts [34] in malaria cases identified during asymptomatic screening in the present study (80% of malaria cases had Ct > 30), suggesting low parasite density across most cases. This low parasite density may be responsible for the high rate of failure of microscopy to detect malaria cases captured by PCR, either due to the parasite density being below the limit of microscopic detection (~50–100 parasites/μL) and/or the increased likelihood of an erroneous microscopic classification. Consequently, molecular methods such as PCR [22] or serological surveillance are required to detect these asymptomatic reservoirs [16,35].

This study has several limitations. First, the analysis was based on routinely collected surveillance data, and additional survey data on risk factors, such as demographic data, adherence to chemoprophylaxis, duration of exposure, and use of personal protective measures, was not available. These factors may play a significant role in malaria exposure and infection risk in military settings. Second, regular screening was not done during the mission, so asymptomatic rates and sub-microscopic rates could only be assessed after returning from deployment. The lack of examination of these factors in the present study precludes us from examining the causes underlying the prophylaxis failure and limits our ability to recommend solutions. These factors should be studied in upcoming UN Peacekeeping Mission deployments to South Sudan in order to more fully identify the risk factors associated with prophylaxis failure and develop solutions to mitigate those risks.

## 5. Conclusions

In conclusion, the findings emphasize the critical role of active post-deployment screening and molecular diagnostics in detecting asymptomatic and sub-microscopic malaria infections among deployed personnel. Without this molecular screening approach, over 60% of soldiers with blood-stage *P. falciparum* parasitemia returning from this deployment would arrive at their home stations untreated, where they could contribute to malaria transmission if the area is suitable, or be at risk for a late diagnosis or misdiagnosis if they are returning to an area where malaria is not commonly diagnosed. The risk of malaria importation is particularly important as many countries that contribute to peacekeeping operations are either non-endemic countries with suitable conditions for malaria transmission, or are endemic countries actively engaged in malaria elimination efforts that would be significantly undermined by the importation of new, rare, or recently eliminated *Plasmodium* species [3,5,7,16] and/or potentially drug-resistant strains [14,32].

## Figures and Tables

**Table 1:** Malaria case rates during UNMISS deployments 2023–2025.

Deployment	UNMISS 2022				UNMISS 2023				UNMISS 2024			
Location	Juba, South Sudan				Juba, South Sudan				Juba, South Sudan			
Dates	March 2022 to July 2023				March 2023 to July 2024				March 2024 to July 2025			
Total personnel	271				270				273			
	Total	Pf	Pv	Po	Total	Pf	Pv	Po	Total	Pf	Pv	Po
Cases in country (all symptomatic)	19	17	1	0	32	28	4	0	34	26	8	0
Cases post-deployment (all asymptomatic)	12	12	0	0	17	17	0	0	29	28	0	1
Microscopic (+)	5	5	0	0	8	8	0	0	8	7	0	1
PCR (+)	12	12	0	0	17	17	0	0	29	28	0	1
Sub-microscopic	7	7	0	0	9	9	0	0	21	21	0	0

## Data Availability

The original contributions presented in this study are included in the manuscript. Further inquiries can be directed to the corresponding author.

## Abbreviations

The following abbreviations are used in this manuscript:

Ct	Number of threshold cycles in a polymerase chain reaction run
PCR	Polymerase Chain Reaction
RDT	Rapid diagnostic test
RTA	Royal Thai Army
UN	United Nations
UNMISS	United Nations Mission in South Sudan
